# Supplementary material for: USP2 Mitigates Reactive Oxygen Species-Induced Mitochondrial Damage via UCP2 Expression in Myoblasts
Source: Int J Mol Sci. 2024 Nov 6;25(22):11936. doi: 10.3390/ijms252211936 (PMC11593688; doi:10.3390/ijms252211936)
Supplement: Supplementary file 1 [file ijms-25-11936-s001.zip › ijms-3157334-supplementary.pdf]

Supplementary Materials

Article title:

USP2 mitigates reactive oxygen species-induced mitochondrial damage via UCP2 expression in myoblasts

Journal name:

International Journal of Molecular Sciences

Author names:

Hiroshi Kitamura, Masaki Fujimoto, Mayuko Hashimoto, Hironobu Yasui, Osamu Inanami

Corresponding author:

Hiroshi Kitamura, Ph.D., D.V.M., D.J.C.L.A.M.

Laboratory of Disease Models, Department of Veterinary Medicine, School of Veterinary Medicine, Rakuno Gakuen University, Ebetsu, Japan

E-mail: ktmr@rakuno.ac.jp

Contents:

1）Table S1

2）Figure S1

Table S1: Sequences of RT-qPCR primers and probes

Sequences of primers and a dual labeled probe for RT-qPCR analysis are shown.

Gene symbol	strand	sequence
Cat	Forward	5'-CACTGAAGATGGTAACTGGGATC-3'
	Reverse	5'-GGATCCTTCAGGTGAGTCTG-3'
Cs	Forward	5'-CCAACCAATCTGCACCCTAT-3'
	Reverse	5'-AATGAGGTCCATGCAGTCCT-3'
Gclc	Forward	5'-CTGCCTCTGTAGATGATAGAACAC-3'
	Reverse	5'-GATGGTCAGGTCGATGTCATTG-3'
Glrx	Forward	5'-GGAAGGTGGTCGTGTTCATC-3'
	Reverse	5'-GTGTTGTTAGTGGCTGTGATGTC-3'
Glrx2	Forward	5'-GGACCTTTGGCTATGTCCTG-3'
	Reverse	5'-TGTAGACTTCCCCCAAAACG-3'
Gsta4	Forward	5'-TGAGAAGATGCAAAAGGATGG-3'
	Reverse	5'-TCCTTCAGGTCCTTCCCATA-3'
Gstm4	Forward	5'-TGGGGAGACAGAGGAAGAGA-3'
	Reverse	5'-GCCCAGGAACTGTGAGAAGA-3'
Gpx1	Forward	5'-ATCAGTTCGGACACCAGGAG-3'
	Reverse	5'-TCACCATTCACTTCGCACTT-3'
Gr1	Forward	5'-GGTGGAAGTCAATGGGAAAA-3'
	Reverse	5'-TCCAGCTGAAAGAAGCCATC-3'
Hmox1	Forward	5'-GATGCTGACAGAGGAACACAAAG-3'
	Reverse	5'-AGGAGCGGTGTCTGGGATG-3'
	Probe	5'-CCCTCACAGATGGCGTCACTTCGTCA-3'
Hprt1	Forward	5'-TCATTATGCCGAGGATTTGG-3'
	Reverse	5'-ACTTTTATGTCCCCCGTTGA-3'
Nfe2l2	Forward	5'-GCAGCATAGAGCAGGACAT-3'
	Reverse	5'-GCTGTCCATTTCTGTCAGTGTG-3'
Nqo1	Forward	5'-CACAGGTGAGCTGAAGGACTC-3'
	Reverse	5'-CTGCAGCTTCCAGCTTCTTG-3'
Ppargc1a	Forward	5'-AACAATGAGCCTGCGAACAT-3'
	Reverse	5'-AAATGAGGGCAATCCGTCTT-3'
Prdx1	Forward	5'-TTTCAGTGATAGAGCCGATGAA-3'
	Reverse	5'-GGTCCCAATCCTCCTTGTTT-3'
Prdx2	Forward	5'-GCTGGGAGTGTCTGTGGACT-3'

	Reverse	5'-TCATTTTTCAACACGCCGTA-3'
Prdx3	Forward	5'-TCATCTTGCCTGGATCAACA-3'
	Reverse	5'-GCACTTTCCAACAGCACTCC-3'
Prdx6	Forward	5'-CATCCTTTTGGGCATGTTG-3'
	Reverse	5'-TGGTGGCAGGGTAGAGGATA-3'
Selenos	Forward	5'-TGTTGTTGTTAAGCGGCAAG-3'
	Reverse	5'-TGCTGTCCCACATTTCAATC-3'
Sod1	Forward	5'-CGGATGAAGAGAGGCATGTT-3'
	Reverse	5'-TTGTTTCTCATGGACCACCA-3'
Sod2	Forward	5'-TCAATGGTGGGGGACATATT-3'
	Reverse	5'-GCTTGATAGCCTCCAGCAAC-3'
Txnrd1	Forward	5'-TGTCACACCGACTCCTCTTG-3'
	Reverse	5'-CCATAGTTGCGCGAGTCTTT-3'
Ucp2	Forward	5'-GGCTCTGGAAGGGACTTCT-3'
	Reverse	5'-AGTGGCAAGGGAGGTXATCT-3'
Ucp3	Forward	5'-TGTTTACTGACAACTTCCCCTGT-3'
	Reverse	5'-GGGCACAAATCCTTTGTAGAAG-3'
Usp2	Forward	5'-CAGGGTCTGGCTGGTCTTC-3'
	Reverse	5'-CTTCCATGAGGGCCGTGT-3'

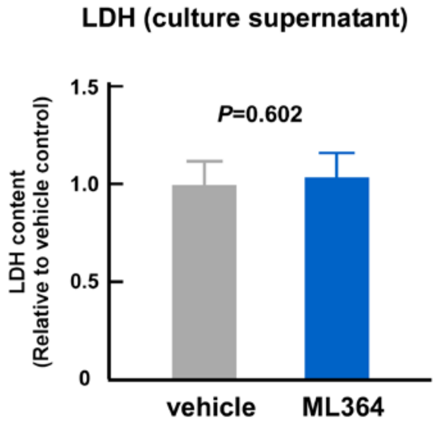

Figure S1: Validation of the toxicity of ML364 to C2C12 cells

C2C12 cells (4 x 10^5 cells/3.5 cm dish) were incubated in the presence of 10 μM ML364 or a vehicle (DMSO, 2 mM) for 8 h. Subsequently, the culture supernatant was collected and lactate dehydrogenase activity was measured using a commercially available kit (Dojindo, Kumamoto, Japan) and an iMark Microplate Absorbance Reader (Bio-Rad, Hercules, CA, USA). Data are expressed as the mean of six wells, normalized to the mean of the vehicle-treated group. The *P*-value shows no difference between the groups.
